# Modulatory Effects of Huoxiang Zhengqi Oral Liquid on Gut Microbiome Homeostasis Based on Healthy Adults and Antibiotic-Induced Gut Microbial Dysbiosis Mice Model

**DOI:** 10.3389/fphar.2022.841990

**Published:** 2022-03-24

**Authors:** Min Gao, Xinhao Duan, Xiang-Ru Liu, Shiyue Luo, Shixin Tang, Hao Nie, Jing Yan, Zhen Zou, Chengzhi Chen, Qi Yin, Jingfu Qiu

**Affiliations:** ^1^ School of Public Health and Management, Chongqing Medical University, Chongqing, China; ^2^ Institute of Life Sciences, Chongqing Medical University, Chongqing, China; ^3^ Dongsheng Lung-Brain Disease Joint Lab, Chongqing Medical University, Chongqing, China

**Keywords:** traditional herbal medicine, Huoxiang Zhengqi, gut microbiota modulation, gut microbiome homeostasis, antibiotic-induced gut microbiota dysbiosis

## Abstract

Traditional herbal medicine (THM) is used worldwide for its safety and effectiveness against various diseases. Huoxiang Zhengqi (HXZQ) is an extensively used Chinese THM formula targeting gastrointestinal disordered gastroenteritis *via* regulating the intestinal microbiome/immuno-microenvironment. However, the specific mechanisms remain largely unexplored, besides as a lifestyle drug, its safety on the gut microbiome homeostasis has never been investigated. In this study, the effects of HXZQ on the gut microbiome of healthy adults were investigated for the first time, and the antibiotic-induced gut microbiota dysbiosis mice model was applied for verification. Based on healthy adults, our results revealed that HXZQ exhibited mild and positive impacts on the bacterial diversity and the composition of the gut microbiome in a healthy state. As for an unhealthy state of the gut microbiome (with low bacterial diversity and deficient compositions), HXZQ significantly restored the bacterial diversity and recovered the abundance of *Bacteroidetes*. In the antibiotic-induced mice model, HXZQ distinctly revived the deficient gut microbial compositions impaired by antibiotics. At the genus level, the abundances that responded most strongly and positively to HXZQ were *Bifidobacterium* in healthy adults and *Muribaculaceae*, *Lactobacillus*, and *Akkermansia* in mice. In contrast, the abundance of *Blautia* in healthy adults, *Enterococcus*, and *Klebsiella* in mice showed inversely associated with HXZQ administration. At last, HXZQ might exhibit an anti-inflammatory effect by regulating the concentration of interleukin-6 in plasma while causing no significant changes in the colon tissue structure in mice. In conclusion, our results elucidate that the safety of HXZQ in daily use further reveals the modulatory effects of HXZQ on gut microbial community structure. These results will provide new insights into the interaction of THM and gut microbiome homeostasis and clues about the safe use of THM as a lifestyle drug for its further development.

## Introduction

The gut microbiome, by interfering with host immunity, digestion, and metabolism, plays an indispensable role in the host's health (Fan and Pedersen, 2021). Accumulating evidence has suggested the intimate connection between the gut microbiota dysbiosis and the pathogenesis of various human diseases, including gastrointestinal diseases, obesity, autism, Parkinson's, cardiovascular disorders, and autoimmune disorders, even tumor development (Fong et al., 2020; Ruff et al., 2020; Weersma et al., 2020; Morais et al., 2021). Moreover, the gut microbiota dysbiosis has been reported to be associated with clinical treatment fails, including but not limited to the chemotherapy and immunotherapy efficacy in cancer treatment and treatments for inflammatory bowel disease (Helmink et al., 2019; Lavelle and Sokol, 2020). Under such circumstances, gut microbiota modulation strategies *via* probiotics, prebiotics, postbiotics, and fecal microbiota transplantation show promising effects as rising stars for the prevention and treatment of various health problems involved in gut microbiota dysbiosis. However, these modulations display interindividual variability. More importantly, the accompanying risks, including adverse effects, personalized efficacies, the transmission of drug resistance genes, or unrecognized pathogens, need to be answered before clinical decisions (Fong et al., 2020; Fassarella et al., 2021). As for other alternative regional medicines or ingredients with long-term safety profiles, the Chinese traditional herbal medicine (THM) has been reported to possess prebiotic-like effects in intestinal homeostasis modulation *via* promoting probiotics proliferation, inhibiting the growth of pathogens and immunomodulation both *in vitro* and *in vivo* (Xie et al., 2011; Chang et al., 2015; Ruan et al., 2015; He et al., 2016; Zhou et al., 2016; Lin et al., 2019).

In targeting gastrointestinal disorders, a classic Chinese THM prescription, named Huoxiang Zhengqi (HXZQ) from Song Dynasty in the early 12th century, has been used to treat functional dyspepsia, gastrointestinal cold, and acute gastroenteritis and also has been applied for relieving heatstroke, nausea, carsickness, and diarrhea in daily life, which is a lifestyle drug commonly used in China (Liu et al., 2014; Zhao et al., 2018). Various *in vitro* and *in vivo* pharmacological studies showed HXZQ and its active ingredients possessing antibacterial, anti-inflammatory, antiallergic, immunity-enhancing, and intestinal mucosal-protective effects (He et al., 2006; Liu et al., 2014; Zhao et al., 2018). Moreover, recent clinical trials have shown that a combination of HXZQ dropping pills with western medicine may have clinical advantages for coronavirus disease 2019 patients in improving clinical symptoms, with gut microbiota maintenance and immunomodulatory effects as underlying mechanisms (Xiao et al., 2020; An et al., 2021). Although its therapeutic effects in curing gastrointestinal disorder-associated diseases have been confirmed by numerous clinical applications, the investigation of the fundamental mode of action is still at its early stage.

In our previous study, based on the lipopolysaccharide-induced acute inflammatory mice model, we found that HXZQ oral liquid exhibited anti-inflammatory effects in both intestine and cortex, with the rejuvenation of intestinal digestive enzymes by pretreatment as an underlying mechanism. These results indicated the gut modulatory and anti-inflammatory activities of HXZQ oral liquid against lipopolysaccharide-induced intestinal disorder. However, in view of the widespread use of HXZQ as a lifestyle drug in the daily life of the healthy population in China, the gut modulatory effects of HXZQ on the healthy population with occasionally intestinal discomfort are still elusive. Thus, in this study, we investigated the gut microbiota modulation of HXZQ in healthy adults, verified the effects, and explored underlying mechanisms in the antibiotic-mediated microbiota dysbiosis mice model. These findings will not only improve our knowledge of its pharmacological basis but also provide new clues about the safety of HXZQ in daily use.

## Materials and Methods

### Study Design and Participants

Huoxiang Zhengqi oral liquid (hereinafter referred to as HXZQ) was purchased from Taiji Group Chongqing Fuling Pharmaceutical Factory Co., Ltd. (Cat Number: 19072656). The formula of HXZQ comprises 11 herbs (presented as taxonomic name, drug name in Chinese, and proportion of per 1,000 ml of the product), including 0.8-ml essential oil (steam distillation) of *Pogostemon cablin* (Blanco) Benth [*Lamiaceae*; Guanghuoxiang-overground part], 80 g of *Atractylodes macrocephala* Koidz. [*Asteraceae*; Baishu-rhizome], 80 g of *Magnolia officinalis* Rehder and E.H.Wilson *Cortex* [*Magnoliaceae;* Houpu-cortex, stir-baked with ginger juice in a ratio of 1:10], 80 g of *Arum ternatum* (Thunb.) Makino [*Araceae*; Banxia-tuber], 0.4-ml essential oil (steam distillation) of *Perilla frutescens* (L.) Britton [*Lamiaceae*; Zisu-fructus], 120 g of *Actaea dahurica* (Turcz. ex Fisch. and C.A.Mey.) Franch. [*Ranunculaceae*; Baizhi-rhizome], 80 g of *Citrus reticulata* Blanco [*Rutaceae*; Chenpi-pericarp], 120 g of *Poria cocos* (Schw.) Wolf [*Polyporaceae*; Fuling-sclerotium], 10-g liquorice extract of *Glycyrrhiza glabra* L. [*Fabaceae*; Gancao-radix et rhizome], and 120 g of *Areca catechu L.* [*Arecaceae*; Dafupi-pericarp], *Zingiber officinale* Roscoe [*Zingiberaceae*; Shengjiang-rhizome]. The detailed proportions of each of the ingredients were cited by Pharmacopoeia of the People's Republic of China, 2020.

Participants older than 18 years were screened out by the following terms: 1) Use of antibiotics within 3 months; 2) use of microbial preparations (i.e., probiotics) and other drugs within 1 month; 3) surgery or invasive treatment or examination; 4) digestive tract symptoms such as diarrhea, bloody stools, and dark stools; 5) adverse allergic reactions; and 6) smoking or drinking alcohol during this program. Finally, 40 participants (19 women and 21 men) were enrolled for the administration of HXZQ. The first-round fecal sample collection was conducted within 24 h (day 0) before the first oral administration of HXZQ (10 ml/day, p.o.), the second-round fecal sample collection was conducted within 24 h after seven consecutive days of oral HXZQ (day 8), and the last round (3rd) fecal sample collection was conducted within 24 h after another seven consecutive days of oral HXZQ (day 15).

### Animals

Seven-week-old C57/BL6J mice (approximately 20–25 g) were purchased from Experimental Animal Center of Chongqing Medical University [Chongqing, China, license numbers: SCXK(Yu)2018-0003]. Mice were allowed to adapt to the experimental housing conditions (temperature maintained at 23 ± 1°C, humidity fixed at 55 ± 10%, 12/12-h light/dark cycle) for 1 week (week 1). Subsequently, mice were randomly divided into four groups (*n* = 16 in each group, half males and half females): 1) the control group, with regular drinking water; 2) the antibiotic cocktail (ABX) group was daily administered (p.o.) with antibiotics (ampicillin 1 g/L, vancomycin 5 g/L, neomycin trisulfate 1 g/L, and metronidazole 1 g/L) in week 2 for gut microbiota dysbiosis modeling (Wang et al., 2012; Morgun et al., 2015) and with regular drinking water for week 3; 3) the HXZQ group, with regular drinking water in week 2 and daily administration of HXZQ (1.3 ml/kg, p.o.) in week 3; 4) the ABX plus HXZQ group (ABXHX group), with antibiotic cocktail gavaged daily in week 2 and daily administration of HXZQ (1.3 ml/kg, p.o.) in week 3. After treatment, animals were killed under anesthesia, and the plasma, colon tissue, and fecal samples were collected. Animal experiments were approved by the Chongqing Medical University Animal Care and Use Committee after careful review.

### 16S Ribosomal RNA Gene Sequencing and Data Analyses

After collection, the fecal samples were stored immediately at −80°C. Total bacterial genomic DNA of fecal samples was extracted using the QIAamp Fast DNA Stool Mini Kit (Omega E. Z.N.A. Soil DNA Kit, United States). The AxyPrep DNA Gel Extraction Kit (Axygenn Biosciences, CA, United States) was used to purify the polymerase chain reaction products. Library construction and 16s ribosomal RNA (rRNA) gene sequencing based on an Illumina MiSeq PE300 platform (Illumina, San Diego, USA) were performed by Shanghai Personal Biotechnology Co. Ltd (Shanghai, China), with primer pairs 338F (5′-ACT​CCT​ACG​GGA​GGC​AGC​AG-3′) and 806R (5′-GGACTACHVGGGTWTCTAAT-3′) (Xu et al., 2016) targeted across the hypervariable region V3-V4 of the bacterial 16S rRNA gene. The sequencing data were analyzed on the free online platform of Majorbio Cloud Platform (www.majorbio.com) as briefly described later. Reads were typically clustered into the operational taxonomic units (OTUs) based upon 16S rRNA gene similarity above 97%. Shannon index and the observed OTU numbers were calculated to evaluate the alpha-diversity of the bacterial community using the Mothur software program (v.1.30.1). Beta-diversity was calculated by Bray–Curtis Principal Coordinate Analysis (PCoA) and partial least squares discriminant analysis (PLS-DA) performed in the R software environment. The linear discriminant analysis effect size (LEfSe) was used to analyze the dominant phylotypes for detecting differences between each group (LEfSe software, https: //hutte nhower.sph.harvard.edu/galaxy/root?tool_id=lefseupload). The relationship between the sample and microbial community was presented using the software Circos-0.67–7 (https: //circos.ca). The PICRUSt was used to predict the functional composition of a microbial community's metagenome. Lastly, Bugbase phenotypic prediction (https://bugbase.cs.umn.edu) was used to determine high-level phenotypes presented in microbiome samples. All sequencing data in this study have been deposited at GenBank under BioProject accession number PRJNA792904 (https://www.ncbi.nlm.nih.gov/bioproject/PRJNA792904).

### Hematoxylin–Eosin Staining

Hematoxylin–eosin (H&E) staining was performed as previously described (Klesney-Tait et al., 2013). Briefly, fresh colonic tissue was immediately immersed in 4% paraformaldehyde solution. The paraffin blocks were sliced into sections, then the sections were deparaffinized in xylene, dehydrated in gradient concentrations of ethanol, and stained with hematoxylin, followed by rinsing in distilled water and staining again with eosin. Lastly, the sections were dehydrated with ethanol, cleared in xylene, and sealed with neutral balsam. Images were acquired using an inverted light microscope (Olympus IX53, Tokyo, Japan).

### Enzyme-Linked Immunosorbent Assay

To detect the immunomodulatory impacts of HXZQ on the peripheral immune of the antibiotic-induced gut microbial dysbiosis mice, the concentrations of four inflammatory cytokines, namely interleukin-1β (IL-1β), interleukin-6 (IL-6), interleukin-10 (IL-10), and tumor necrosis factor-α (TNF-α) in plasma were measured using a QuantiCyto enzyme-linked immunosorbent assay (ELISA) kit (Neobioscience Co. Ltd, China), according to the manufacturer's instructions. The absorbance at 450 nm was read with a microplate reader (Thermo Fisher Scientific Inc., Waltham, MA, United States).

### Statistical Analysis

All data from the questionnaire were tallied using Epidata. Quantitative information was analyzed by SPSS Statistics 21 (Armonk, NY, United States), and nonquantitative information was assigned for the same analysis. Pearson correlation was used to analyze the correlation of data. One-way analysis of variance, Student *t*-test, nonparametric Kruskal–Wallis test, or Wilcoxon rank-sum test was used in the data analysis when appropriate (footnoted in the legend of each figure). Data analysis of ELISA was performed using software GraphPad Prism 5. *p*-values ≤ 0.05 indicated statistical significance.

## Results

### Basic Information and Diet Habits of the Participants

In view of basic information of volunteers (Table 1), the age was between 19 and 26 (average: 23.62 ± 2.50) years old for men and between 23 and 30 (average: 24.58 ± 1.64) years old for women. The body mass index data indicated that all participants were within the normal range. The items involved in living and eating habits indicated that participants follow a healthy lifestyle and routine activities, such as basically no drinking or smoking and basically keeping regular meals. The details are shown in Supplementary Table S1.

**TABLE 1:** Basic information and diet habits of participants. Data were reported as mean ± SEM.

Indicator	Average	Male	Female
Age	23.03 ± 2.59	21.62 ± 2.50	24.58 ± 1.64
Body mass index	21.11 ± 2.29	22.35 ± 2.17	19.74 ± 1.53
Score^a^			
Smoking	3.68 ± 0.66	3.37 ± 083	3.95 ± 0.21
Drinking	3.65 ± 0.77	3.37 ± 0.96	3.90 ± 0.44
Breakfast	3.33 ± 0.86	3.95 ± 0.22	2.63 ± 0.76
Diet	1.90 ± 0.30	1.90 ± 0.30	1.89 ± 0.31
Tasting	3.20 ± 1.42	4.14 ± 1.31	2.16 ± 0.76
Staple food	2.08 ± 0.42	2.10 ± 0.30	2.05 ± 0.52
Food match	1.75 ± 0.44	1.81 ± 0.40	1.68 ± 0.48
Drinking water	2.00 ± 0.75	2.19 ± 0.70	1.79 ± 0.63
Fruit	2.65 ± 0.70	2.90 ± 0.70	2.36 ± 0.60
Defecation	2.00 ± 0.00	2.00 ± 0.00	2.00 ± 0.00
Sleeping	1.55 ± 0.55	1.55 ± 0.55	1.47 ± 0.61

a Scoring Criteria.

Smoking and Drinking: 1, Every day; 2, Occasionally; 3, Already quitting; 4, and Never.

Breakfast: 1, No breakfast; 2, Seldom; 3, Usually; 4, and Every day.

Diet: 1, Irregularly; 2, and Regularly.

Tasting: 1, greasy; 2, Spicy; 3, Sweet; 4, Salty; 5, and Light.

Staple food: 1, Only rice and flour; 2, Preference for rice flour or coarse-grain potatoes; 3, Rice flour and coarse grains and potatoes in equal amounts.

Food match: 1, Other situations; 2, Half meat and half vegetarian.

Drinking water: 1, 400–600 ml 1 day; 2, 600–800 ml 1 day; 3, 800–1000 ml 1 day; 4, Over 2000 ml 1 day.

Fruit: 1, Never; 2, Seldom; 3, Usually; 4, and Every day.

Defecation: 1, Once every 3 days or three times a day; 2, and Once every 2 days or twice a day.

Sleeping: 1, Frequent late nights; 2, Occasional late nights; 3, and No late nights.

### Huoxiang Zhengqi Improved Gut Microbial Diversity of Healthy Adults With Healthy or Sub-healthy State Gut Microbiome

HXZQ oral liquids were administrated daily to 40 participants for two consecutive weeks. The fecal samples were collected and sequenced at time points before administration (day 0), after administration (day 15), and among administration (day 8). The fecal microbial community profiles were clustered into OTUs at a threshold of 97% of 16s rRNA gene sequence similarity for further bacterial diversity analyses. For alpha-diversity, the Shannon diversity index and the observed OTU numbers were compared, and there was no significant difference between each time point at any taxonomic level. Compared with the average level, 10 samples were screened out for the abnormally change trends and/or the low initial alpha-diversity (Supplementary Table S2). Predictably, the regular living and eating habits of a healthy person largely referred to a healthy gut microbial community structure. Thus, we speculated that these 10 individuals involved in gut microbiota dysbiosis with poor diversity due to the individual-dependent differences were possibly related to their personal genotyping and environmental factors, including drug or food allergic history, antibiotic use, smoking history, etc. (Supplementary Table S1). Thus, these 10 individuals were excluded for subsequent analyses for a better perspective of the bacterial composition changes under HXZQ of donors with healthy gut microbial community structure (*n* = 30).

**FIGURE 1:**
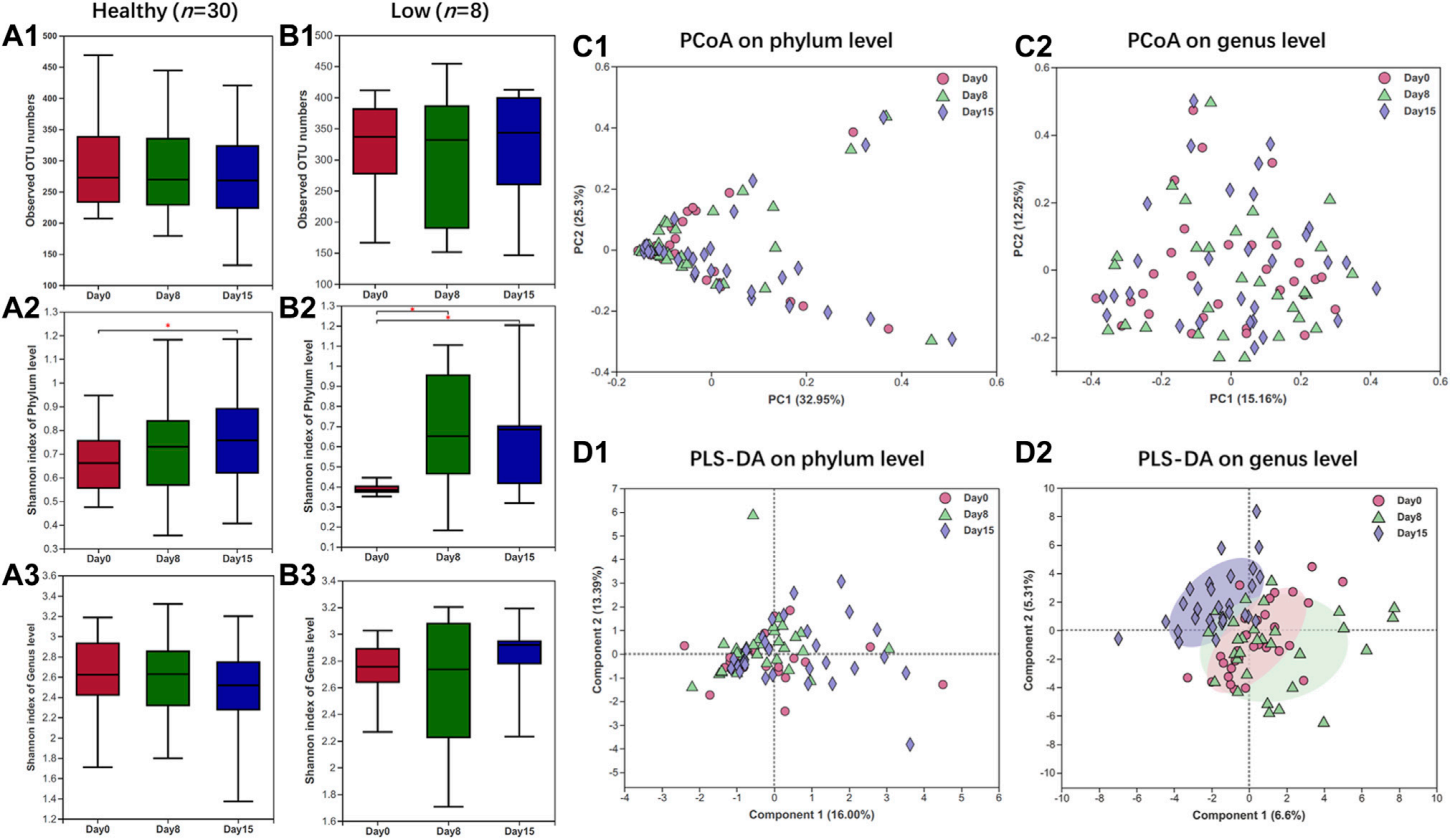
Gut microbial diversity analyses of healthy adults under HXZQ administration for 2 weeks. **(A)** Alpha-diversity based on observed OTU numbers and Shannon diversity of donors with healthy state of gut microbiome (*n* = 30). **(B)** Alpha-diversity based on observed species (richness) and Shannon diversity of donors with low diversity of gut microbiome (*n* = 8). **(C)** Bray–Curtis principal coordinate analysis of gut microbiota of donors with healthy state of gut microbiome (*n* = 30). **(D)** Partial least squares discriminant analysis of gut microbiota of donors with healthy state of gut microbiome (*n* = 30). * *p*-value < 0.05.

**FIGURE 2:**
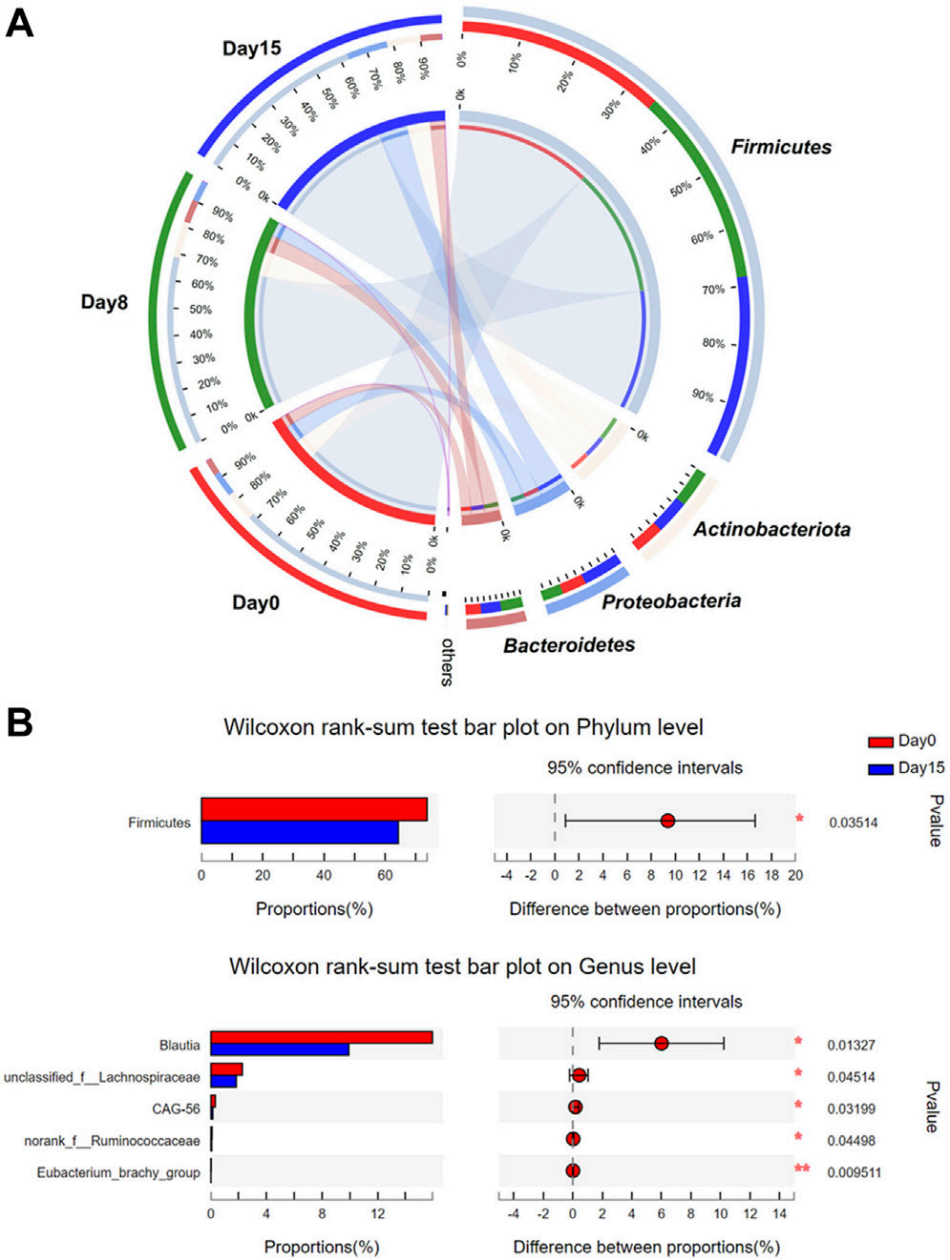
Component comparison analyses of bacterial community structure after HXZQ administration on donors with healthy state of gut microbiome (*n* = 30). **(A)** Circos analysis was used to visualize bacterial community structure at each time point. **(B)** Visual representation of *p*-values obtained from comparison of an individual in group days 0 and 15. Wilcoxon rank-sum test was applied. * *p*-value < 0.05; **p < 0.01.

Overall, based on donors with a healthy state of the gut microbiome (*n* = 30), there was no significant difference in the alpha-diversity after a 7-day administration of HXZQ, instead of a slight elevation after a 14-day administration (day 0 *versus* 15, *p* < 0.05, Figure 1A). As for donors with gut microbiota dysbiosis, designated low initial alpha-diversity (*n* = 8), the Shannon diversity index of days 8 and 15 at the phylum level was significantly higher than day 0 (Figure 1B), which indicated the restorative effect of HXZQ on gut microbial composition with deficiency. These results indicated that the HXZQ exhibited no harmful impacts but a mildly positively modulatory effect on the gut microbial composition. Subsequently, PCoA and PLS-DA analyses (beta-diversity) on the phylum level also showed no obvious change of gut microbial diversity under HXZQ administration (Figures 1C,D). In contrast, PLS-DA analysis on the genus level showed that (Figures 1D, 2) samples of day 15 clustered with group members and separated from samples of days 8 and 0, whereas samples of days 0 and 8 gathered together, which indicated that HXZQ exhibited slightly modulatory effects on gut microbial community structure. Both alpha- and beta-diversities indicated that HXZQ exhibited mildly and positively modulatory impacts on gut microbiome homeostasis in both healthy and unhealthy states of the gut microbiome in adults. These results further indicated the safety of HXZQ in daily use in view of gut microbial community structure.

### Integrated Analysis of Bacterial Profiles of Healthy State Gut Microbiome in Adults Under Huoxiang Zhengqi Administration

At the phylum level, *Firmicutes* was the predominant bacteria in the intestinal of three time points, followed by *Actinobacteria*, *Proteobacteria*, and *Bacteroidetes* (Figure 2A). After the administration of HXZQ, the abundance of *Firmicutes* was observed to have gradually decreased (day 0 *versus* 15, *p* < 0.05), accounting for 73.6% (day 0), 69.9% (day 8), and 64.3% (day 15), respectively. In contrast, the abundances of *Proteobacteria* and *Bacteroidetes* were observed to increase, accounting for 7.7% (day 0), 13.6% (day 8), and 15.1% (day 15) of *Proteobacteria* and 5.9% (day 0), 8.5% (day 8), and 7.6% (day 15) of *Bacteroidetes* (day 0 *versus* 15, *p* = 0.06), respectively. There were no distinct differences at each taxonomic level in the gut microbial composition of days 0 and 8. At the genus level, *Blautia* showed a gradually decreased abundance (15.9% on day 0, 13.6% on day 8, and 9.9% on day 15, *p* < 0.05, Figure 2B), whereas *Bifidobacterium* (8.5% on day 0, 9.8% in day 8, and 10.5% in day 15) showed a gradually increasing trend of abundance (Supplementary Table S3). Additionally, LEfSe revealed no significant differences after HXZQ administration (Supplementary Figure S1). Considering the underlying functional compositions in each gut microbial community predicted based on 16S rRNA gene profiles, the cluster of orthologous groups (COG) analyses indicated no significant differences between each time point (Supplementary Figure S2). These results further confirmed the mild impacts of HXZQ on the gut microbiome of healthy adults that also indicated that the administration of HXZQ oral liquid as a daily health regulation for relieving occasional discomforts was safe to use.

### Huoxiang Zhengqi Recovered the Gut Microbial Diversity in Antibiotic-Induced Gut Microbiota Dysbiosis Mice Model

**FIGURE 3:**
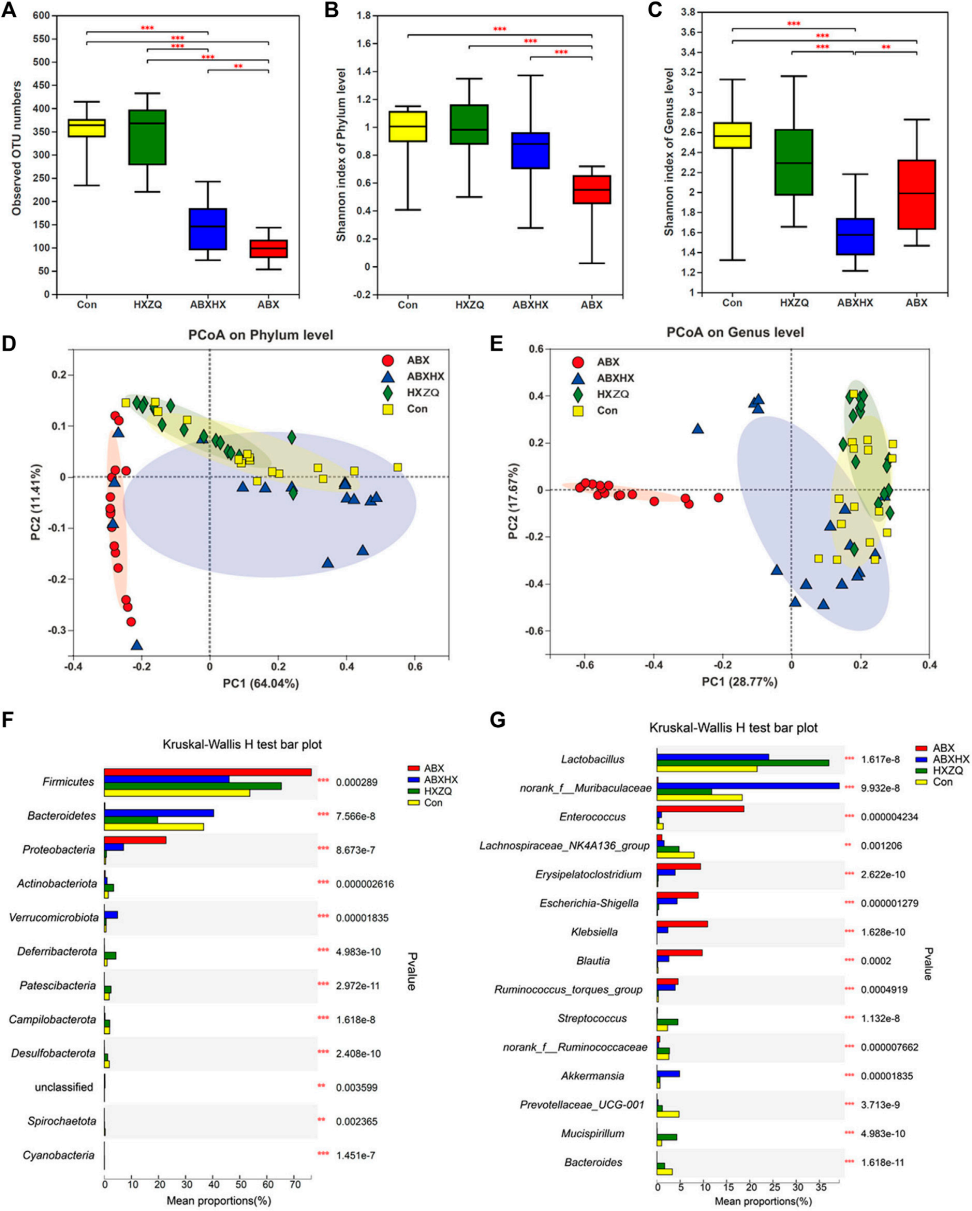
Bacterial diversity analyses and comparison of bacterial community structure after HXZQ administration in antibiotic-induced gut microbiota dysbiosis mice model (*n* = 16 in each group). Alpha-diversity based on observed OTU numbers **(A)** and Shannon diversity on phylum level **(B)** and genus level **(C)** were compared in four group mice. Bray–Curtis principal coordinate analysis on phylum level **(D)** and genus level **(E)** of gut microbiota. **(F,G)** Visual representation of *p*-values obtained from comparison of bacterial abundance on phylum and genus level between four groups. Kruskal–Wallis H test was applied. * *p*-value <0.05; **p < 0.01; ***p < 0.001.

To investigate the modulatory effects of HXZQ on gut microbial composition, the gut microbiota dysbiosis mice model induced by antibiotic cocktail (ABX) treatment was used. As results showed in Figures 3A–C, alpha-bacterial diversity (the observed OTU numbers and the Shannon index on phylum level) of mice in the ABX group decreased sharply, far below those of mice in the control and HXZQ groups. Meanwhile, the bacterial diversity of mice in the ABX plus HXZQ (ABXHX) group was recovered compared with those in the ABX group, which indicated that HXZQ exhibited restorative effects on gut microbiota dysbiosis. Moreover, comparing the HXZQ group with the control group, there was no significant difference in bacterial diversity in accordance with the population data. As for beta-diversity, Bray–Curtis PCoA on both phylum level and gene level showed that group HXZQ and group control clustered together with group ABXHX, which surrounded nearby, away from group ABX (Figures 3D,E). These results indicated that the gut microbial community structure disturbed by antibiotics could be restored by HXZQ administration.

### Huoxiang Zhengqi Significantly Modulated the Gut Microbial Composition in Antibiotic-Induced Gut Microbiota Dysbiosis Mice Model

The modulatory effects of HXZQ on gut microbiome homeostasis also were confirmed by the changes in the gut microbial compositions in the mice model. On the phylum level (Figure 3F, Supplementary Figure S3A), compared with control, antibiotics induced a marked increment of *Firmicutes* (*p* = 0.001), accompanied by the increment of *Proteobacteria* (*p* < 0.001) and the approximate disappearance of *Bacteroidetes* (*p* < 0.001) in the ABX group mice, which indicates that the gut microbial dysbiosis model was successfully constructed and hard to recover by itself in the short term. Meanwhile, mice in the HXZQ group showed a decreased abundance of *Firmicutes* (*p* = 0.022) but an increased abundance of *Bacteroidetes* (*p* = 0.011), compared with control (Supplementary Figures S3A3). Compared to the ABX group, mice in the ABXHX group showed a significantly decreased abundance of *Firmicutes* (*p* < 0.001) and *Proteobacteria* (*p* = 0.002) but an increment abundance of *Bacteroidetes* (*p* < 0.001, Supplementary Figures S3A4). At the genus level (Figure 3G, Supplementary Figures S3B), *Muribaculaceae*, *Lactobacillus*, and *Akkermansia* were the dominant species with significantly increasing abundance in the ABXHX group, compared with the ABX group (*p* < 0.001, Supplementary Figures S3B4). These results observed in the mice model were in accordance with the population data described earlier. Meanwhile, the LEfSe analysis revealed biologically consistent differences in each group (LDA >3.0), with *Klebsiella* as a significantly enriched species in the ABX group (*p* < 0.001), whereas *Muribaculaceae* as a significantly enriched species in the ABXHX group (*p* values < 0.05) (Supplementary Figures S4).

### Huoxiang Zhengqi Was Predicted to Affect Metabolic and Pathogenic Functions of Gut Microbiota

**FIGURE 4:**
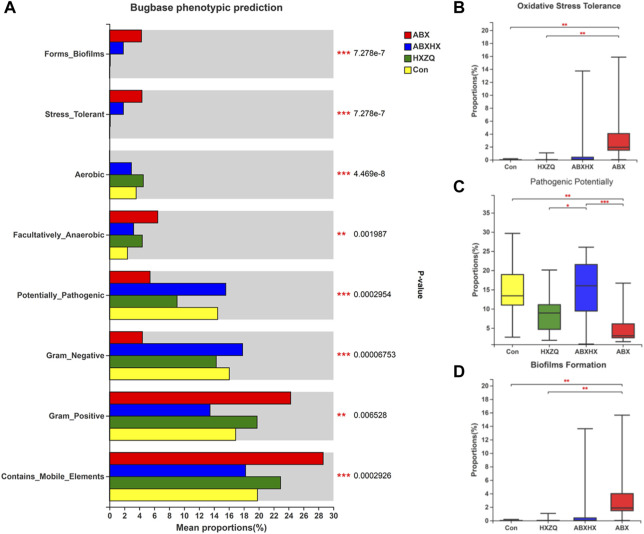
Bugbase phenotypic prediction of gut microbiota in mice model. **(A)** Comparisons between four groups of all phenotypes. **(B)** Oxidative stress tolerance. **(C)** Pathogenic potentially. **(D)** Biofilm formation. Kruskal–Wallis H test was applied. * *p*-value <0.05; **p < 0.01; ***p < 0.001.

The relative abundance of COG function classification showed that the highest represented function of “Carbohydrate transport and metabolism” was predicted to be alleviated in the gut microbiome of the ABX group and restored in those of the ABXHX group to the level of control (Supplementary Figure S5). Moreover, based on the Bugbase phenotypic prediction (Figure 4), the directly pathogenic related aspects, namely the microbial oxidative stress tolerance and the ability of biofilm formation, increased in the ABX group, whereas the pathogenic potentially decreased compared with control (*p* values <0.001). Notably, the pathogenic potentially showed an alleviation in the HX group compared with the control. These results indicated that HXZQ could modulate the unstable or unhealthy state of antibiotic-induced gut microbiota dysbiosis.

### Hematoxylin–Eosin Staining and Enzyme-Linked Immunosorbent Assay Revealed Huoxiang Zhengqi Might Modulate the Immune Microenvironment

**FIGURE 5:**
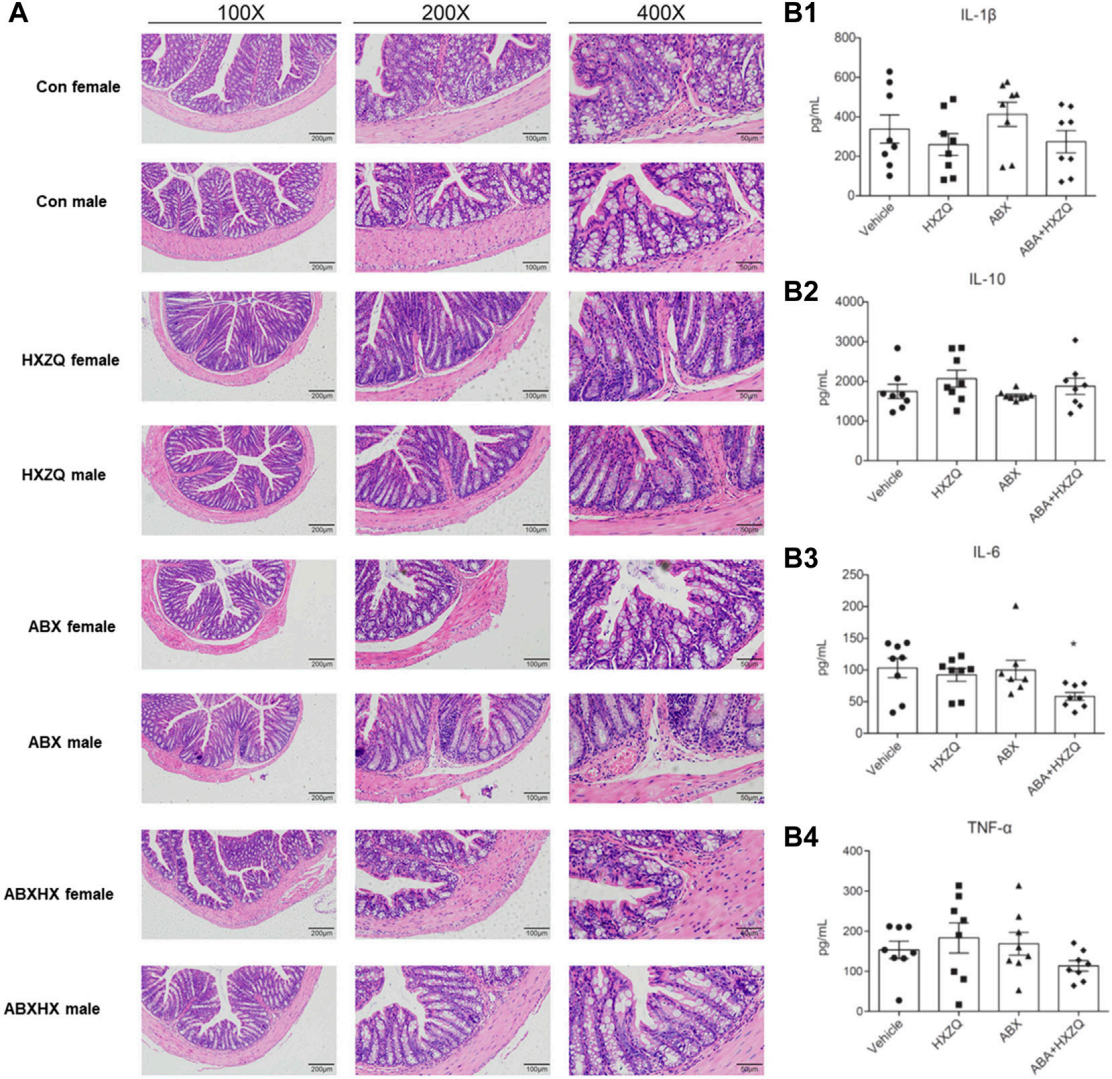
Effects of HXZQ on immuno-microenvironment in colon tissue of mice. **(A)** Hematoxylin–eosin staining was used to detect morphological changes in structure of colon. **(B)** Concentrations of four inflammatory indicators in plasma were detected using ELISA kit analyses. Data were reported as mean ± SEM. Statistical analysis was performed using an independent Student *t*-test. *p-value < 0.05, represented significant difference.

To investigate the underlying mechanisms of the modulation of HXZQ on gut microbiota dysbiosis, H&E staining of colon tissue and ELISA of four inflammatory cytokines (IL-1β, IL-6, IL-10, and TNF-α) in plasma were conducted to detect the immunomodulatory impacts of HXZQ on the peripheral immune microenvironment. As shown in Figures 5A, the concentration of IL-6 was reduced in ABX gut dysbiosis mice under HXZQ administration (*p* < 0.05). Although without statistical significance, there were similar decreased trends in the concentrations of IL-1β and TNF-α. Meanwhile, in colon tissue structure, there were sporadic crypts with inflammation viewed in the ABX group (Figure 5B), and no sex-dependent differences were observed. These results indicated that HXZQ might exhibit immunomodulatory effects on the colon immune microenvironment.

## Discussion

Innumerable THMs have been proven to possess therapeutic efficacy against a broad spectrum of diseases in both historic and modern sciences (Lin et al., 2019). Artemisinin from the Chinese herb *Artemisia apiacea* against malaria was awarded the 2015 Noble Prize in Physiology or Medicine. Paclitaxel from herb Pacific yew is an efficient anticancer drug worldwide. Sodium oligomannate (GV-971), a marine algae-derived oligosaccharide, is proposed as a safe and well-tolerated candidate in anti-Alzheimer's disease therapy by several clinical trials (Xiao et al., 2021). However, the development and application of traditional medicine are staggered on account of complex ingredients with an uncharted and intricate mode of action. On the basis of the therapeutic effects of THM, it seems to be rational to consider safety as an equally important factor of its clinical decisions to face the dilemma of efficient herb medicines with uncharted mechanisms. In other words, THMs with both therapeutic effects and safety are worth promoting in clinical, rather than persistently sticking to the mechanism investigations before use. Fortunately, relying on the blooming development of gut microbiome sequencing, the interactions of THM and intestinal microbiota come into view and could provide cogent and straightforward interpretations about the underlying mechanisms of THM treatment. However, the investigations are at an early stage and require further elucidation.

The Chinese herbal formula, HXZQ, possesses an exceptional safety profile over hundreds of years, targeting functional dyspepsia, gastrointestinal disorders, and daily heatstroke and nausea. It has over one billion CNY (approximately 0.16 billion USD) of annual sales revenue in the Chinese market and has been registered and listed in 14 countries and regions, including the United States of America, Canada, Singapore, Thailand, etc. However, as a lifestyle drug for daily discomforts, the modulatory effects of HXZQ on adults with healthy or sub-healthy gut microbiome have never been investigated. In this study, our results revealed that HXZQ established mildly and positively modulatory effects on gut microbial community structure in healthy adults (*n* = 30), with no distinct differences in both alpha- and beta-diversities after 14 days of HXZQ administration. Notably, HXZQ significantly restored the gut microbial diversity of adults with unhealthy gut microbiome (poor and sole bacterial structure, *n* = 8) by reducing the abundance of *Firmicutes* but elevating the abundance of *Bacteroidetes*. An increasing number of studies have evidenced that the healthy state (stability and resilience) of the human gut microbiome is intensely relevant to human health. The unhealthy state of the gut microbiome involves a significant reduction in diversity, potentially leading to a deficiency of profitable bacteria and the expansion of pathogenic microbes in the spare ecological niches in the intestinal. Thus, we verified the modulatory effects of HXZQ in the antibiotic-induced gut microbiota dysbiosis mice model. In the present study, antibiotics induced profound depletion of gut microbiota compositions, resulting in a reduced bacterial diversity and a significantly increased abundance of pathogenic bacteria *Klebsiella*. ABX-treated mice showed an extremely high abundance of *Firmicutes* but near disappearance of *Bacteroidetes*. After 7 days of HXZQ administration, the abundance of *Bacteroidetes* was revived in the ABXHX group, which further confirmed the modulatory effect of HXZQ on gut microbiome homeostasis.

At the genus level, the population data revealed that the species primarily affected by HXZQ involved *Blautia* and *Bifidobacterium*. *Blautia* is a butyric and acetic acid production *Firmicutes*, with the potential to maintain or to improve the status of diseases related to metabolic syndrome (Ozato et al., 2019). Meanwhile, *Bifidobacterium* is a lactic and acetic acid production probiotic, possessing protection and maintenance of a healthy gut microenvironment. In the present study, *Blautia* showed a gradually decreasing trend of abundance (15.9% on day 0, 13.6% on day 8, and 9.9% on day 15, *p* < 0.05), whereas *Bifidobacterium* showed a gradually increasing trend of abundance (8.5% on day 0, 9.8% on day 8, and 10.5% on day 15). Regarding the same substances of these two bacteria, we speculated that HXZQ administration conferred the survival of *Bifidobacterium* to modulate the balance of short-chain fatty acid producers in the intestinal. Animal experiments in the present study further confirmed the positively modulatory effects of HXZQ on the gut microbiome and intestinal immune microenvironment. *Muribaculaceae*, a gut bacterium contributes to the propionate production, which was reported to correlate with an extended life span (Smith et al., 2019); *Lactobacillus*, a commonly used probiotic (Nowak et al., 2019), and *Akkermansia*, a gut bacterium possessing immunomodulatory effect to improve the survival of cancer patient with checkpoint blockade immunotherapy (Routy et al., 2018), were observed as the dominant species with significantly increasing abundance in the ABXHX group. Our results might provide an additional perspective about the therapeutic effects of HXZQ: the enriched abundances of these profitable bacteria by HXZQ administration might be beneficial to the gut microbiota homeostasis.

Last but not least, to link its gut microbial modulation with immunophenotypic characterizations, we investigated the immunomodulatory effects of HXZQ in the mice model. In accordance with gut microbial composition, HXZQ exhibited no significant differences in the concentration of pro- and anti-inflammatory cytokines in plasma yet showed reducing trends in pro-inflammatory factors (IL-1β, IL-6, and TNF-α), comparing the ABXHX group with the ABX group. The H&E staining analysis also revealed that HXZQ exhibited negligible effects on the colon tissue structure yet reduced the occurrence of inflammation in crypts in the ABXHX group compared with the ABX group. These results indicated the mild modulatory effects of HXZQ on the colon immune microenvironment and the ability to regulate the unhealthy state into a healthy state. Last, we need to emphasize the limitations of our work. The underlying molecular mechanisms about the way how HXZQ modulating of gut microbial compositions of HXZQ need to be addressed in more detail. The changes of both concentrations and compositions of bacterial metabolisms need to be detected and connected to the bacterial diversity shifts under HXZQ treatment. It also remains to enroll more participants to sketch its modulatory effects on the gut microbiome on a large scale, as well as to enroll patients with gastrointestinal disordered gastroenteritis to survey the therapeutic effects of HXZQ in long-term treatment. At best, we made to first step in these directions. As a lifestyle drug targeting gut microbiome homeostasis modulation, its impacts on the gut microbial community of a healthy population, who frequently use HXZQ as a relieving medicine for daily discomfort, should be an important criterion for its safety evaluation. In this view, the present study provides new clues for its safe use in daily life.

In summary, the present study used healthy population data and an antibiotic-induced gut microbiota dysbiosis mice model to profile the modulatory effects of HXZQ oral liquid on gut microbiome homeostasis. Firstly, HXZQ could elevate both alpha- and beta-bacterial diversities of the gut microbiome in both healthy or unhealthy states while exerting no impacts on the colon tissue structure and the gut immune microenvironment. Noteworthy, HXZQ possessed the ability to restore the imbalance gut microbial community *via* increasing the abundance of beneficial bacteria (i.e., *Bifidobacterium*, *Lactobacillus*, *Akkermansia*, etc.) to positively modulate the gut microbiome homeostasis. To our best knowledge, this is the first time investigating the safety of HXZQ in view of the healthy gut microbiome in adults, combined with an antibiotic-induced gut microbiota dysbiosis mice model. Further investigations will be proceeded to help us understand the mode of action of HXZQ deeply. The present study illustrates the modulatory effects of HXZQ on the gut microbiome and will provide new insights into the development of THM in the treatment of gastrointestinal diseases.

## Data Availability

The original contributions presented in the study are publicly available. This data can be found here: https://www.ncbi.nlm.nih.gov/bioproject/PRJNA792904.
