# Supplementary material for: Modulatory Effects of Huoxiang Zhengqi Oral Liquid on Gut Microbiome Homeostasis Based on Healthy Adults and Antibiotic-Induced Gut Microbial Dysbiosis Mice Model
Source: Front Pharmacol. 2022 Mar 24;13:841990. doi: 10.3389/fphar.2022.841990 (PMC8987308; doi:10.3389/fphar.2022.841990)
Supplement: Supplementary file 1 [file Presentation1.pdf]

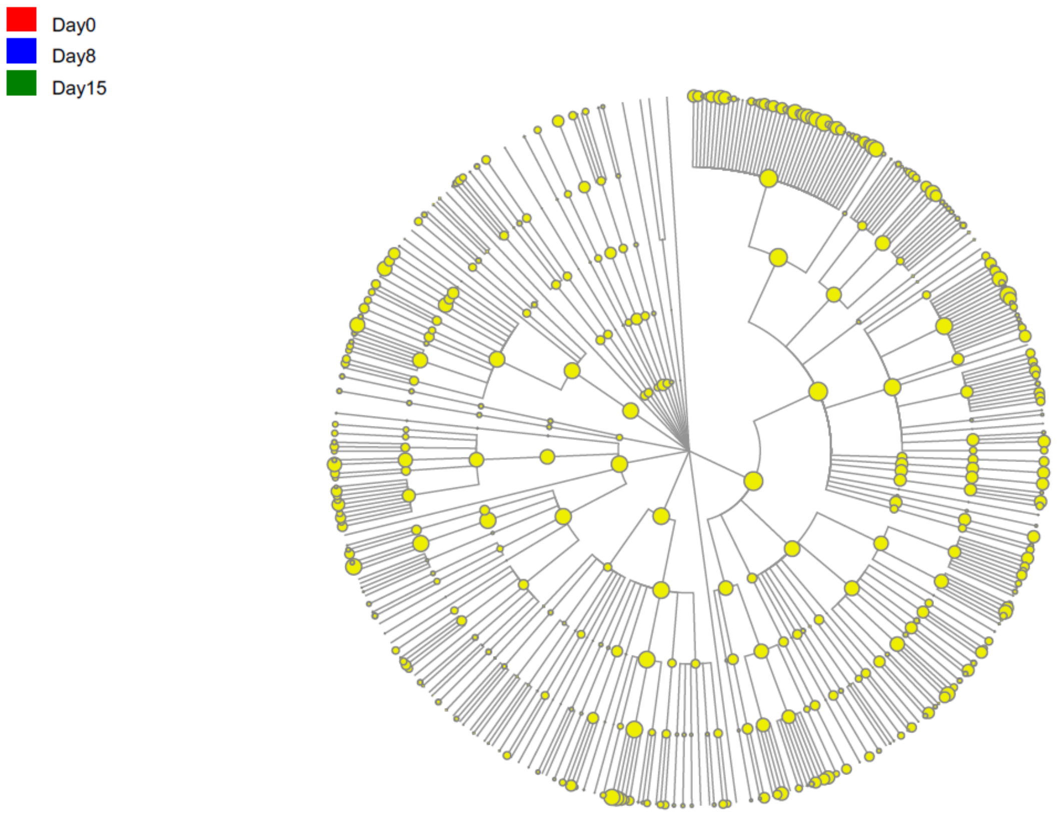

Figure S1 Linear discriminant analysis (LDA) coupled with effect size (LEfSe) used to detect the bacterial clades showing none statistically significant and biologically consistent differences of bacterial components in each time point during HXZQ administration of adults with healthy state of gut microbiota (LDA score > 3.0, *n*=30).

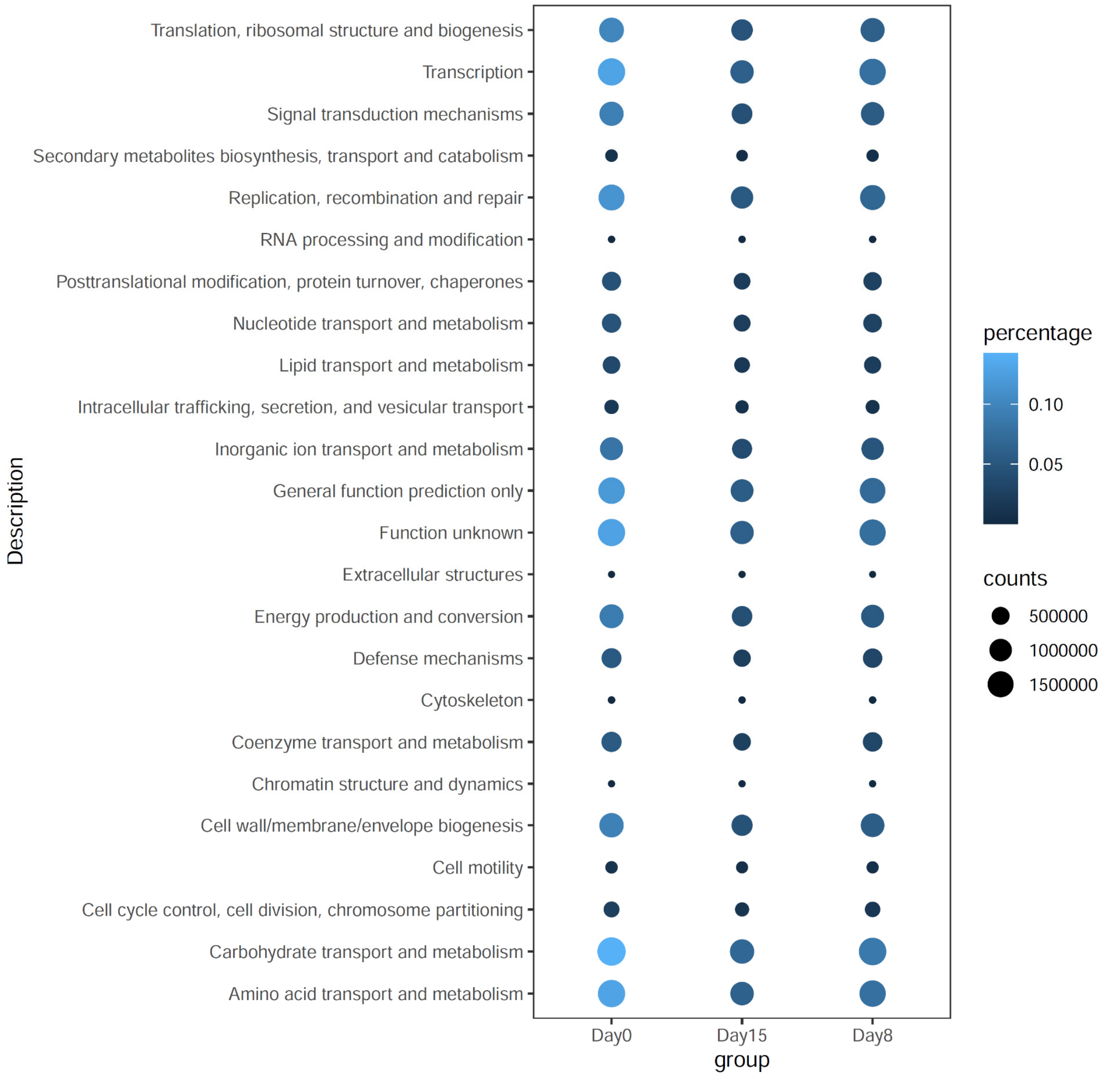

Figure S2 The relative abundance of COGs function classification of gut microbial community predicted based on 16S rRNA gene profiles, based on adults with healthy state of gut microbiome (*n*=30).

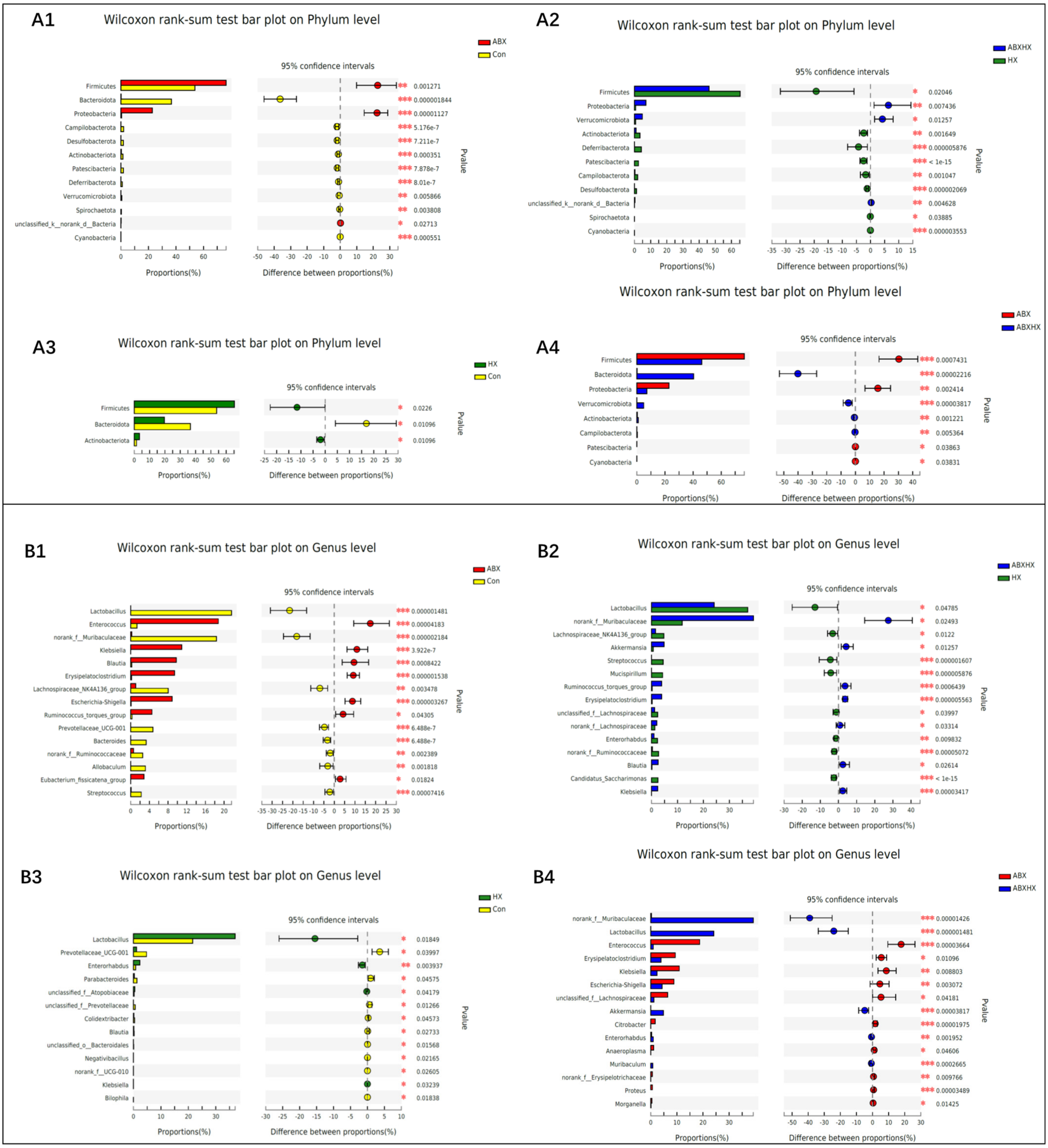

Figure S3. Visual representation of p values obtained from comparison of bacterial abundance on phylum and genus level between four groups. Kruskal-Wallis H test was applied. *, represented p value < 0.05.

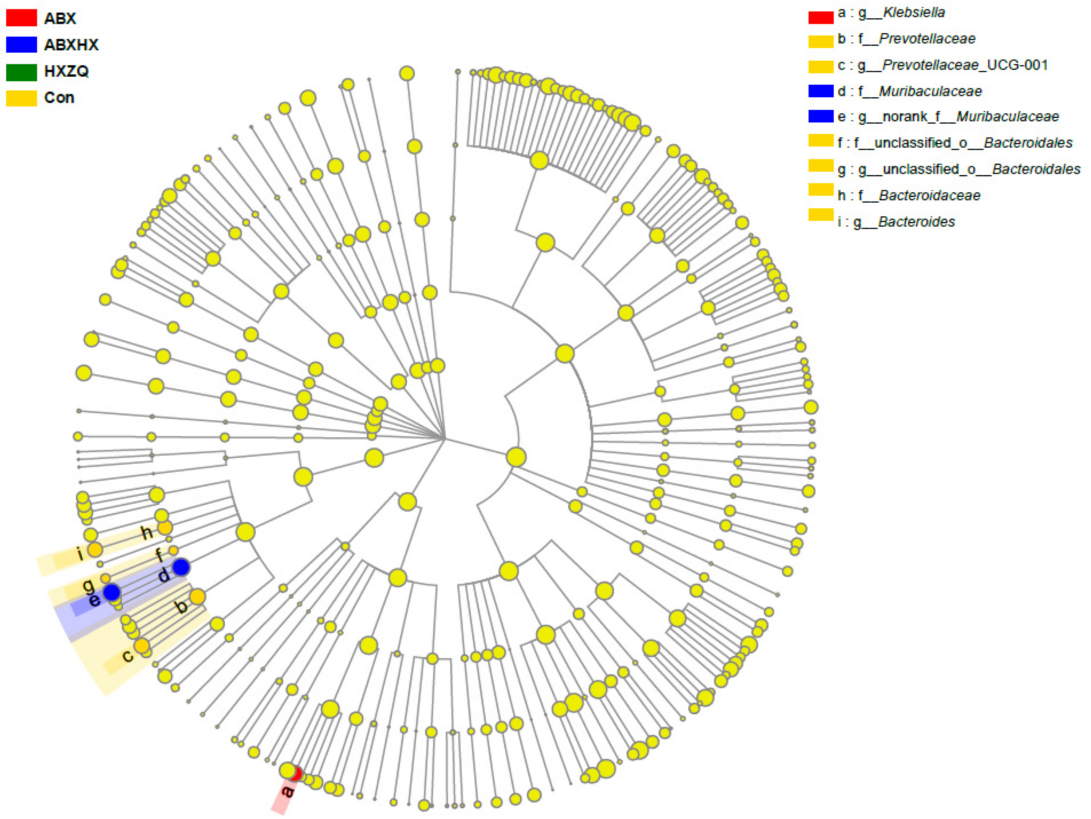

Figure S4 Linear discriminant analysis (LDA) coupled with effect size (LEfSe) used to detect the biologically consistent differences in each group in mice (LDA score > 3.0).

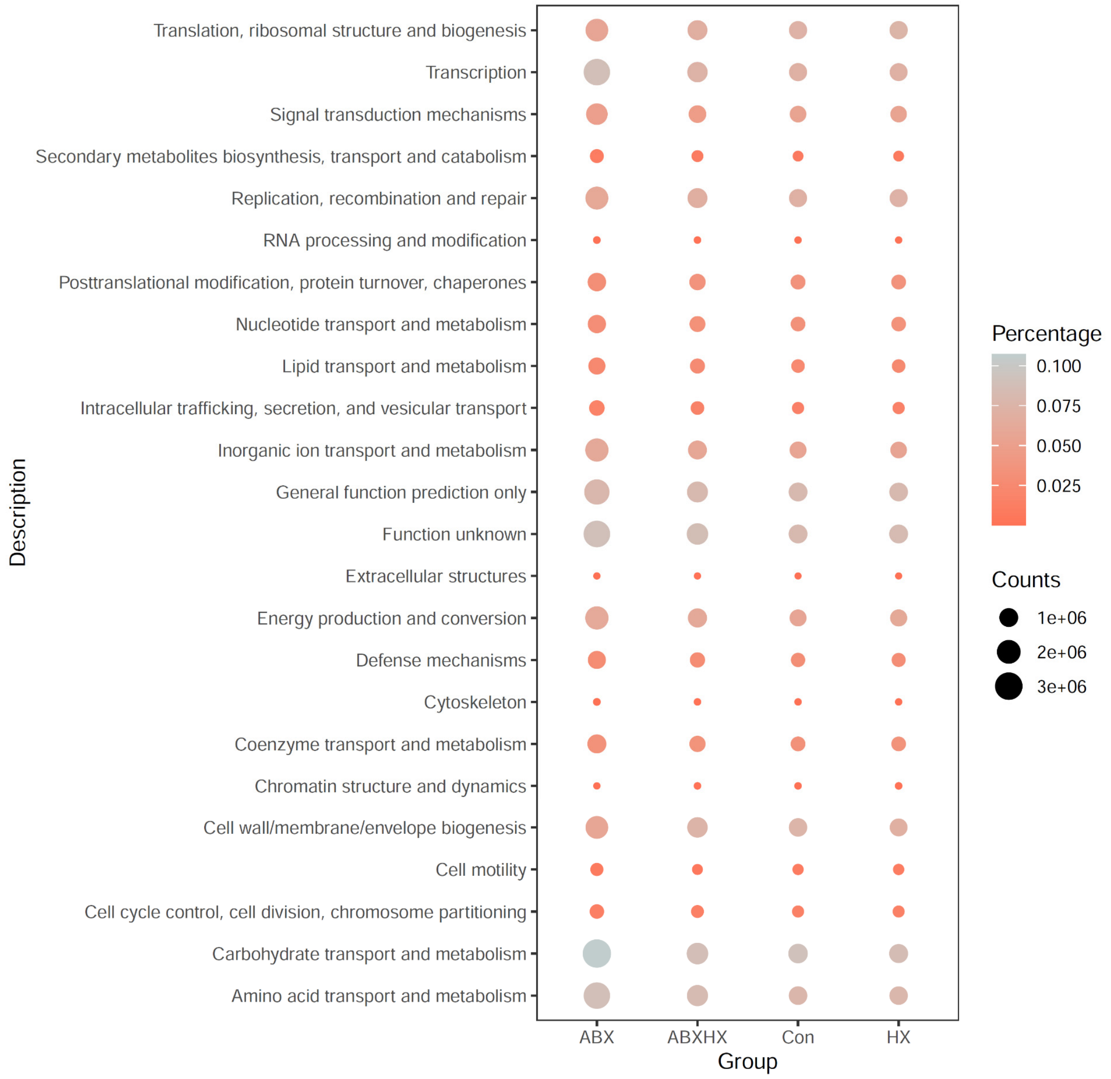

Figure S5. The relative abundance of COGs function classification of gut microbial community predicted based on 16S rRNA gene profiles of mice model.
