# Supplementary material for: Modulatory Effects of Huoxiang Zhengqi Oral Liquid on Gut Microbiome Homeostasis Based on Healthy Adults and Antibiotic-Induced Gut Microbial Dysbiosis Mice Model
Source: Front Pharmacol. 2022 Mar 24;13:841990. doi: 10.3389/fphar.2022.841990 (PMC8987308; doi:10.3389/fphar.2022.841990)
Supplement: Supplementary file 3 [file Table2.pdf]

Table S2. Samples with unhealthy state of gut microbiome identified according to the abnormal or low Shannon diversity in phylum level in this study.

Sample\Es	sobs	shannon	ace	chao	Sample\Es	sobs	shannon	ace	chao	Sample\Es	sobs	shannon	ace	chao
Day0					Day8					Day15				
A1_1	9	0.51507	16.49625	12	A1_2	8	0.699009	17.56444	9	A1_3	8	0.577339	8	8
A10_1	11	0.44395	11.5156	11	A10_2	10	0.744033	10.85875	10	A10_3	9	0.694483	9	9
A11_1	8	0.536145	8.525377	8	A11_2	8	0.567756	8.451556	8	A11_3	8	0.937132	0	11
A12_1	5	0.672825	5	5	A12_2	7	0.389959	7.54519	7	A12_3	8	0.619723	8.744898	8
A13_1	8	0.750827	8	8	A13_2	9	0.868725	9.393519	9	A13_3	8	1.185266	8	8
A14_1	8	0.891147	8.748047	8	A14_2	8	0.953774	0	8	A14_3	8	0.945855	8.703704	8
A15_1	10	0.714485	18.68967	11.5	A15_2	7	0.574513	7	7	A15_3	7	0.893312	0	7
A16_1	10	0.381609	11.4032	10	A16_2	9	0.182351	9	9	A16_3	9	0.357225	9.444444	9
A17_1	7	0.601086	0	10	A17_2	8	0.367421	10.35556	9	A17_3	7	0.733945	7	7
A18_1	10	0.350537	10.5	10	A18_2	9	0.556446	12.66667	10	A18_3	9	0.437237	9.5125	9
A19_1	7	0.382382	7.784	7	A19_2	8	0.358454	9.944904	9	A19_3	8	0.317054	0	11
A2_1	8	0.653913	12.24524	8.5	A2_2	8	0.596403	14.78	9	A2_3	7	0.545868	7	7
A20_1	8	0.862055	8	8	A20_2	9	0.847807	9.497031	9	A20_3	8	0.61261	8.9375	8
A21_1	7	0.41062	7	7	A21_2	11	0.499761	11	11	A21_3	9	0.705147	9	9
A22_1	8	0.554778	9.111111	8	A22_2	7	0.788184	7	7	A22_3	7	0.681267	8	7
A23_1	10	0.738726	13.20338	10.5	A23_2	8	0.787224	9.58612	8	A23_3	7	0.818647	7	7
A24_1	9	0.3975	9.557516	9	A24_2	9	1.103788	10.12	9	A24_3	9	0.699244	18.80331	10
A25_1	8	0.665522	8	8	A25_2	8	0.54517	14.26883	8.5	A25_3	8	0.829476	8	8
A26_1	7	0.94566	8.854595	7	A26_2	7	0.879705	7	7	A26_3	8	0.88133	9	8
A27_1	8	0.591382	8.643125	8	A27_2	6	0.283178	6	6	A27_3	7	0.481716	7	7
A28_1	6	0.769898	6	6	A28_2	6	0.80455	7.872	6	A28_3	6	0.779636	6	6
A29_1	5	0.738087	0	5	A29_2	6	0.732992	6	6	A29_3	8	0.80958	11	8.5
A3_1	9	0.835416	9	9	A3_2	9	0.984614	12	9.5	A3_3	9	0.789008	9	9
A30_1	10	0.932847	10	10	A30_2	8	0.999943	8	8	A30_3	9	1.061189	9	9
A31_1	6	0.581854	6	6	A31_2	6	0.72691	0	6	A31_3	6	0.84486	6	6
A32_1	7	0.473975	7.527111	7	A32_2	6	0.812884	0	6	A32_3	5	0.669252	5	5
A33_1	7	0.727346	7.619141	7	A33_2	9	1.182201	9.54519	9	A33_3	7	0.996129	8.801749	7
A34_1	6	0.548234	7	6	A34_2	6	0.476682	6	6	A34_3	7	0.585363	8	7
A35_1	8	0.775878	8.395044	8	A35_2	8	0.769018	9.877508	8	A35_3	8	0.706497	9.41983	8
A36_1	6	0.352231	6	6	A36_2	10	0.917039	10.3757	10	A36_3	7	0.674209	8.125	7
A37_1	7	0.385291	8.111111	7	A37_2	10	1.0612	28.4758	11.5	A37_3	10	1.20314	10.704	10
A38_1	8	0.657237	9	8	A38_2	6	0.596588	6	6	A38_3	6	0.688387	6	6
A39_1	9	0.529622	10.94766	9.5	A39_2	7	0.67301	16.80331	8	A39_3	9	1.126054	9	9
A4_1	7	0.499988	7	7	A4_2	6	0.689288	6	6	A4_3	7	0.406251	7.524527	7
A40_1	7	0.707232	7	7	A40_2	8	0.763583	9.12	8	A40_3	7	0.453098	7.5	7
A5_1	9	0.511195	9	9	A5_2	10	0.96297	10	10	A5_3	10	1.005297	10	10
A6_1	9	0.756847	12.45423	10	A6_2	8	0.736828	9.832031	8	A6_3	8	0.538138	0	8
A7_1	9	0.559444	9.606736	9	A7_2	6	0.354945	6	6	A7_3	8	0.49708	9.032922	8
A8_1	7	0.643444	7	7	A8_2	8	0.455254	8.565972	8	A8_3	7	0.657592	8.111111	7
A9_1	7	0.579962	7.619141	7	A9_2	9	0.479672	9	9	A9_3	8	0.620929	8.9375	8
**average**	7.875	**0.61566**	8.663933	8.125	**average**	7.9	**0.69435**	9.287501	8.1125	**average**	7.775	**0.72664**	7.707575	7.9625
(grey)	represented abnormal trend of Shannon index;				(light red)	represented gut microbiota with low Shannon index.								

***Samples with abnormal (n=2) or low (n=8) Shanon index compared with the average level in each time point (mainly considered the initial diversity), were identified as sub-healthy or unhealthy state of gut microbiota (n=10), and excluded in the analysis of healthy adults with healthy state of gut microbiome.**
